# Biomechanical mechanisms of early gait training on knee cartilage degeneration after anterior cruciate ligament reconstruction: a study protocol

**DOI:** 10.3389/fspor.2026.1833258

**Published:** 2026-06-24

**Authors:** Ruiqin Dang, Yiqun Lu, Ziren Zhou, Zheng Zhou, Na Cui, Yuanyuan Yu, Si Zhang, Yingfang Ao, Hongjie Huang, Jianquan Wang, Shuang Ren

**Affiliations:** 1 Department of Sports Medicine, Peking University Third Hospital, Institute of Sports Medicine of Peking University, Beijing, China; 2 Beijing Key Laboratory of Research and Translation for Drugs and Medical Devices in Precision Diagnosis and Treatment of Sports Injuries, Beijing, China; 3 Engineering Research Center of Sports Trauma Treatment Technology and Devices, Ministry of Education, Beijing, China

**Keywords:** anterior cruciate ligament reconstruction, cartilage degeneration, gait biomechanics, gait retraining, weight-loss treadmill

## Abstract

**Objective:**

The primary objective of this study is to investigate the effects and biomechanical mechanisms of early gait retraining (GRT) on knee cartilage degeneration following anterior cruciate ligament reconstruction (ACLR).

**Methods:**

This study is a single-blind, parallel-group randomized controlled trial. A total of 60 participants scheduled for primary unilateral ACLR will be recruited and randomly allocated to either the experimental group (conventional rehabilitation plus gait retraining) or the control group (conventional rehabilitation only). Gait retraining will commence at 3 weeks post-surgery, lasting for 6 weeks with a frequency of 2–3 sessions per week, each lasting 15–20 min. Data will be collected at baseline (pre-intervention) and at 3 months, 6 months, 1 year, and 2 years postoperatively. Assessment will include three-dimensional gait analysis, surface electromyography (sEMG), isokinetic muscle strength testing, functional magnetic resonance imaging (fMRI), Y-balance test, single-leg hop test, the International Knee Documentation Committee (IKDC) subjective knee evaluation form, Lysholm knee score, and Tegner activity scale. The primary outcome measure is the peak knee flexion moment during walking. Secondary outcomes include cartilage T1*ρ*/T2 values, muscle activation patterns, and subjective functional scores. A repeated-measures analysis of variance (ANOVA) will be used to evaluate the intervention effects on the outcome measures.

**Results:**

Not applicable.

**Conclusion:**

Early gait retraining using a lower-body positive pressure (anti-gravity) treadmill after ACLR is hypothesized to significantly improve postoperative gait biomechanics. Furthermore, early GRT may optimize standard rehabilitation protocols, potentially preventing or delaying cartilage degeneration. This approach holds clinical significance for restoring normal gait and preventing secondary injuries in individuals with sports-related injuries.

**Clinical Trial Registration:**

https:register.clinicaltrials.gov, identifier NCT06368544

## Introduction

Anterior cruciate ligament (ACL) rupture is a common and severe sports-related knee injury, accounting for approximately 50% of knee sports injuries (1). ACL rupture can lead to joint instability, disrupt the biomechanical homeostasis of the joint, and impair athletic performance (2, 3).

Arthroscopic anterior cruciate ligament reconstruction (ACLR) is currently the preferred clinical treatment for ACL injuries, effectively restoring the structural stability of the knee joint. However, the re-rupture rate after ACLR exceeds 10% (4, 5), and the risk of developing knee osteoarthritis within 10 years post-surgery remains as high as 50% (6). Abnormal gait biomechanics are considered a significant contributing factor to the development of knee osteoarthritis (7–9). Given that walking is a highly repetitive daily activity, long-term abnormal gait patterns can lead to abnormal stress distribution on articular cartilage (10–12). Over time, this may result in secondary lesions such as cartilage damage and meniscal tears, thereby accelerating cartilage degeneration and the onset and progression of osteoarthritis (13–15).

Current standardized postoperative rehabilitation protocols primarily focus on range of motion, muscle strength, balance, coordination, and functional exercises. Previous studies have found that even when patients regain muscle strength and range of motion to levels sufficient for return-to-sport, abnormal gait biomechanics often persist (10, 16, 17). This indicates that current standardized rehabilitation protocols may be insufficient to fully restore normal gait biomechanics after ACLR, and these persistent abnormalities may adversely influence long-term joint health. Therefore, early gait retraining (GRT) is particularly important. GRT involves the use of various biofeedback modalities to target and modify specific aberrant movement patterns, aiming to restore pre-injury gait patterns (18–20). However, no studies have yet explored the impact of early GRT on gait biomechanics after ACLR.

In summary, there is a lack of evidence regarding the effect of early gait retraining on cartilage degeneration after ACLR. Therefore, this study aims to investigate the effects of early postoperative gait retraining on knee cartilage degeneration and its underlying biomechanical mechanisms through a randomized controlled trial, with the goal of providing a theoretical basis for optimizing postoperative rehabilitation protocols after ACLR.

## Methods

### Study design

**Figure 1:**
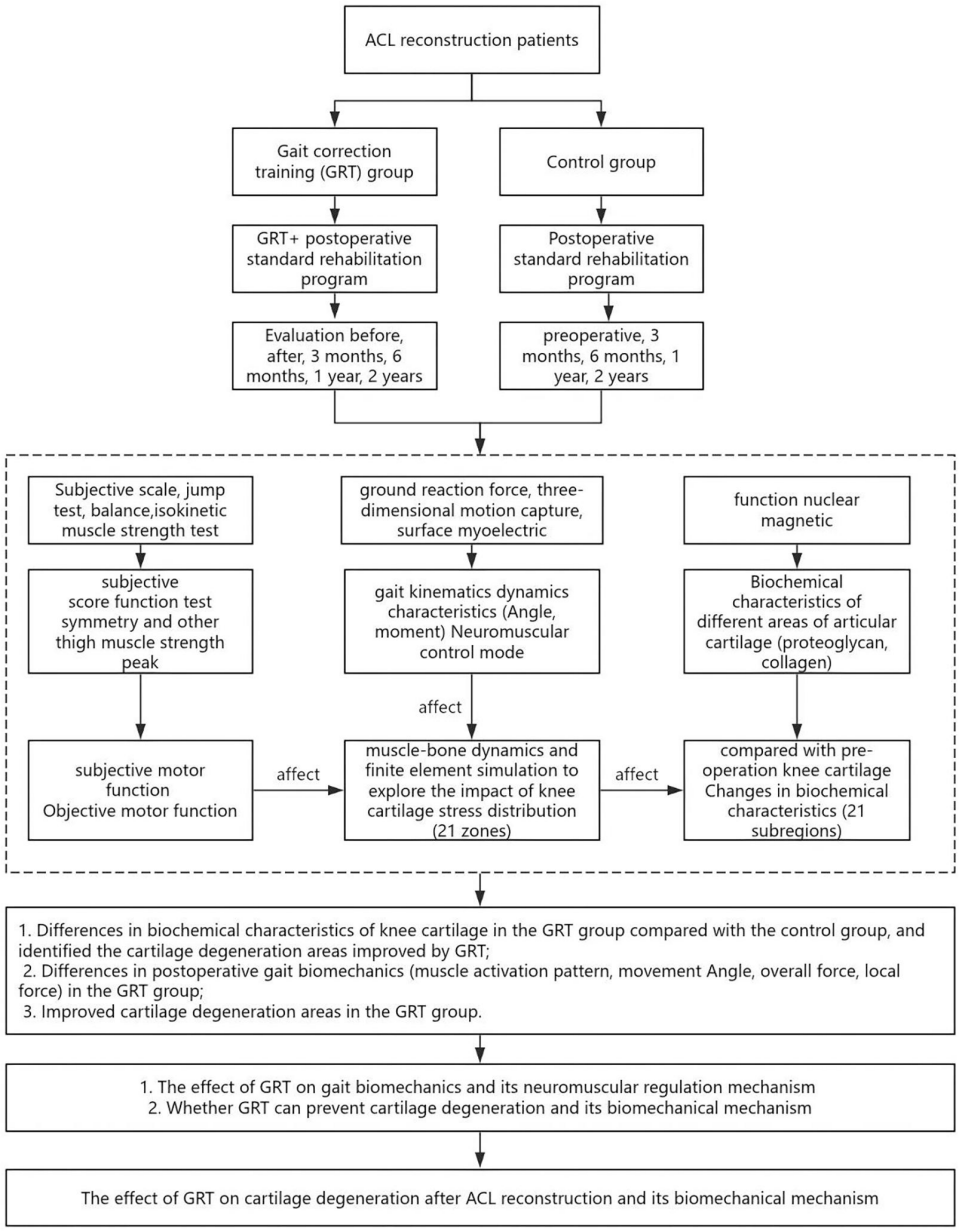
Technical roadmap for the study protocol.

This study is a prospective randomized controlled trial. The experimental group will undergo gait training in addition to standard rehabilitation, while the control group will receive only standard rehabilitation. Both groups will be assessed at baseline, 3 months, 6 months, 1 year, and 2 years postoperatively (Figure 1).

### Participants and recruitment

#### Sample size calculation

The primary outcome measure is the peak knee flexion moment during walking, a key biomechanical indicator of cartilage degeneration (21). A significant difference in this measure will be considered statistically meaningful. Based on preliminary results, the mean peak knee flexion moment before training in ACLR patients is 0.20 BW·BH, with a standard deviation of 0.09. After training, the mean peak knee flexion moment is 0.28 BW·BH, with a standard deviation of 0.11 Nm/kg/m. Using the formula for sample size estimation for two independent samples, $n=\frac{2\sigma^{2}{\left(Z_{\alpha /2}+Z_{\beta }\right)}^{2}}{\delta^{2}}$, with *α* = 0.05 and *β* = 0.2,among $\sigma =\sqrt[{2}]{\frac{\sigma_{1}^{2}+\sigma_{2}^{2}}{2}}$, $\delta =\bar{X_{1}}-\bar{X_{2}}$, the required sample size for each group was calculated to be 25.Accounting for a 20% dropout rate, 30 participants per group will be recruited. The required sample size corresponds to a Cohen's d of 0.8, indicating a large effect size.

#### Inclusion and exclusion criteria

A total of 60 patients scheduled for ACLR will be recruited. After enrollment, participant information will be entered into the RED Cap system, and participants will be randomly assigned to two groups (experimental group, *n* = 30; control group, *n* = 30) using a mixed block randomization method. This is a single-blind randomized controlled trial; participants will not be informed of their group assignment or the intervention details prior to the intervention. Baseline information and data will be collected preoperatively. After the study procedures have been fully explained and understood, written informed consent will be obtained from all participants at the Department of Sports Medicine, Peking University Third Hospital. All data collection and interventions will be conducted at the same department.

Inclusion Criteria: (1) age 18–35 years; (2) diagnosis of ACL rupture confirmed by MRI; (3) first-time unilateral ACL rupture scheduled for reconstruction at our institution; (4) body mass index (BMI) within the normal range of 18.5–23.9 kg/m^2^ (to exclude the confounding effect of obesity on joint loading); (5) time since injury less than 6 months; (6) affected knee has passed the acute phase, with no significant swelling, pain, inflammation, or range of motion limitation, and basic joint mobility is restored.

Exclusion Criteria: (1) history of musculoskeletal injury or surgery on the contralateral lower limb; (2) time since ACL injury 6 months; (3) cartilage injury with Outerbridge grade III or IV; (4) concurrent meniscal tear requiring meniscal repair during ACLR; (5) concomitant severe injury to the posterior cruciate ligament, medial collateral ligament, or lateral collateral ligament; (6) presence of metabolic syndrome (obesity, dyslipidemia, diabetes, etc.), immune system diseases affecting articular cartilage, or severe cardiovascular or cerebrovascular diseases; (7) unwillingness to receive the allocated treatment.

### Outcome measures

#### Measurement procedures

Participants will complete baseline information and subjective functional scoring scales (including basic information, IKDC2000 subjective knee function score, Tegner activity level score, Lysholm knee score, pain VAS score, and dominant side questionnaire). Subsequently, bilateral knee MRI (including functional MRI), three-dimensional gait biomechanics, isokinetic muscle strength, and lower limb balance tests will be conducted. All tests will be performed at baseline, 3 months, 6 months, 1 year, and 2 years postoperatively.

#### Gait analysis

A 10-camera three-dimensional motion capture system (Vicon) will be used to collect three-dimensional kinematic data during walking at a sampling rate of 100 Hz. Ground reaction forces will be collected synchronously using embedded force plates at a sampling rate of 1,000 Hz. Synchronization between the force plates and the motion capture system will be achieved via a hardware trigger signal. Surface electromyography (EMG) will be used to assess muscle activation levels of the quadriceps, hamstrings, tibialis anterior, and gastrocnemius during walking at a sampling rate of 2,000 Hz. EMG electrodes will be placed according to the SENIAM (Surface ElectroMyoGraphy for the Non-Invasive Assessment of Muscles) recommendations, positioned over the muscle belly and aligned with the muscle fiber direction. Gait biomechanical analysis will be performed using Visual 3D software (C-motion, USA) to calculate three-dimensional kinematics and kinetics of the hip, knee, and ankle joints. Additionally, an Anybody musculoskeletal multibody dynamics model (18 rigid bodies, 92 muscles) will be used to calculate overall knee joint contact forces, anterior-posterior and shear forces, and individual muscle forces using inverse dynamics.

### Basic motor function tests

#### Isokinetic muscle strength test

Isokinetic strength of the knee extensors and flexors will be assessed using a dynamometer (Con-Trex MJ, Fitzmann, Germany). Participants will be securely strapped at the trunk and thigh. The knee will be moved from 90° to 20° flexion (and back) at angular velocities of 60 °/s, 180 °/s, and 300 °/s for concentric and eccentric contractions, following standardized protocols.

#### Y-balance test

Participants will stand on a single leg with the great toe aligned with the starting line on the testing platform. The other foot will be used to push the testing board as far as possible in the anterior, posteromedial, and posterolateral directions. The farthest distance reached (accurate to 0.5 cm) will be recorded for each direction, with three repetitions.

#### Single-leg hop test (to be performed at 6 months, 1 year, and 2 years post-surgery)

This includes the single-leg hop, triple single-leg hop, diagonal triple single-leg hop, and 6-meter timed hop. Two formal tests will be performed per participant, and the best result will be recorded. A rest interval of 30 s will be provided between tests.

### Lysholm knee score

Lysholm is a quantitative score for daily symptoms and exercise capacity of the knee joint. This scoring system contains eight questions. The total score ranges from 0 to 100, with higher scores indicating better knee function. According to the score results, the functional status of the knee joint of the patient can be evaluated. More than 95 points are excellent, 94–85 points are good, 84–65 points are fair, and less than 65 points are poor (22).

### IKDC 2,000 subjective knee score

IKDC 2000 knee subjective rating scale is a widely used tool to evaluate knee function and symptoms, especially for patients after knee injury or surgery (23).The scale assesses the performance of the knee joint in daily activities through patient self-report. The overall score ranges from 0 to 100, with higher scores indicating better knee function.

### Tegner activity level score

The Tegner rating scale is a tool commonly used to assess the degree of athletic injury and functional recovery to assess the ability to perform athletic activities after knee injury. The Tegner rating scale is graded on a scale from 0 to 10 based on the level of motor activity participants engage in and the gradual increase in demands (24).

### Functional MRI

**Figure 2:**
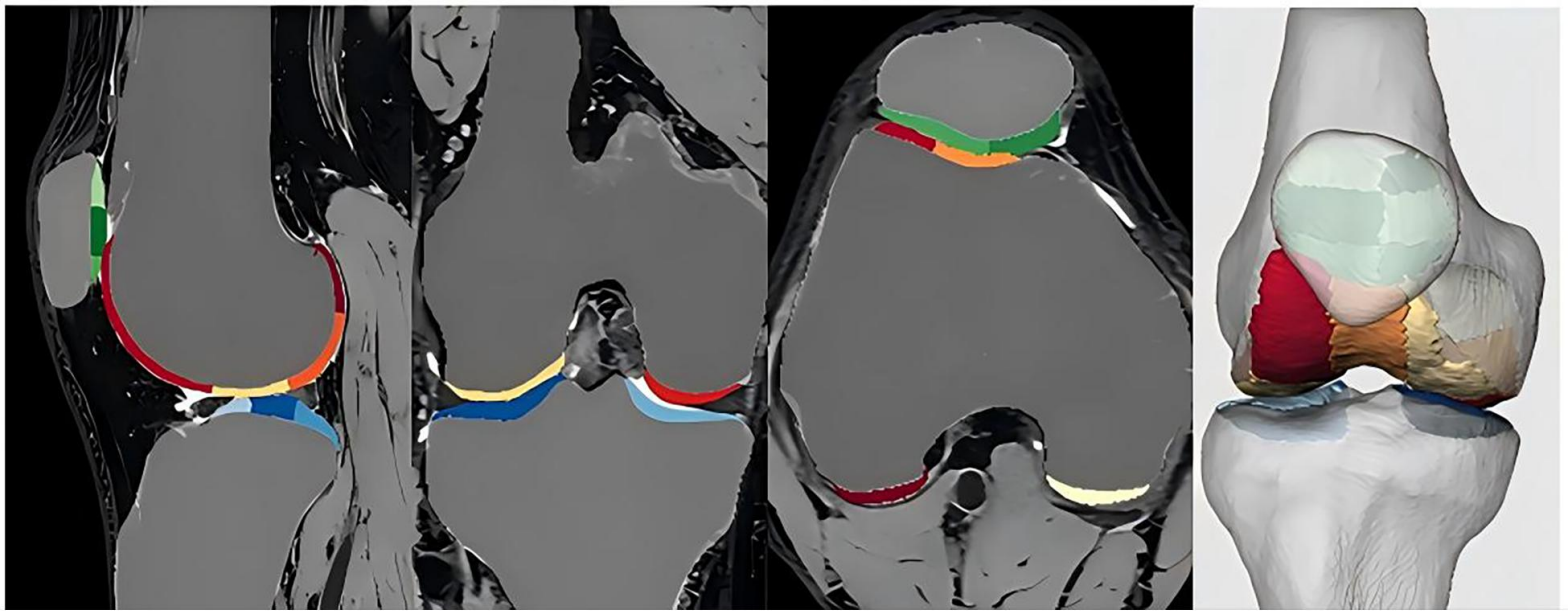
Schematic representation of cartilage partitions in the knee joint. AI segmentation + manual fine tuning in 21 zones of knee cartilage and VR segmentation effect.

Early cartilage degeneration may not be detectable on conventional MRI due to the absence of morphological changes; therefore, functional MRI techniques will be used to assess cartilage relaxation times. A GE 750 W 3.0 T MRI scanner will be used to scan bilateral knees. The protocol includes T1*ρ* and T2 mapping sequences, which reflect cartilage biochemical characteristics (collagen and proteoglycan content). Increases in these values indicate a time-dependent decline in collagen and proteoglycan content, signifying early cartilage degeneration (25–27).The T2 mapping sequence will be performed using a 3D multi-echo fast spin-echo sequence (TR/TE = 9.3/3.7 ms, echo train length = 8). The T1*ρ* sequence will use a spin-lock pulse (locking frequency = 500 Hz, locking time = 10 ms). Other parameters include: FOV = 16 cm, matrix = 256 × 256, slice thickness = 1 mm, voxel size = 0.625 × 0.625 × 1 mm^3^, number of slices = 32, and fat suppression technique (SPECIAL). The regions of analysis will include the medial and lateral femoral condyles, femoral trochlea, medial and lateral tibial plateaus, and patellar cartilage, as illustrated in Figure 2.

### Postoperative gait intervention

#### Experimental group

Participants in the experimental group will undergo standard postoperative rehabilitation as outlined below. In addition, starting from 3 weeks post-surgery, they will concurrently receive a 6-week gait retraining program using a lower-body positive pressure (LBPP) system (Anti-Gravity Treadmill, AlterG). On days when the gait retraining intervention is performed, the walking training component of the standard postoperative rehabilitation protocol will be omitted. After the gait retraining session, patients will continue with their home-based rehabilitation as per the standard protocol.

The anti-gravity treadmill (AlterG) comprises a lower-body positive pressure (LBPP) chamber and a visual feedback screen. During walking, pneumatic pressure unloads a percentage of body weight, thereby reducing knee joint loading and potentially alleviating pain. The LBPP treadmill has been shown to reduce knee pain in patients with knee osteoarthritis (28). It provides a relatively safe environment for walking, offering advantages over traditional therapy for the restoration of walking ability, gait correction, and balance improvement (28–30). It integrates the three key elements of walking—loading, stepping, and balance—thereby facilitating the formation of normal gait patterns (29, 31).

**Figure 3:**
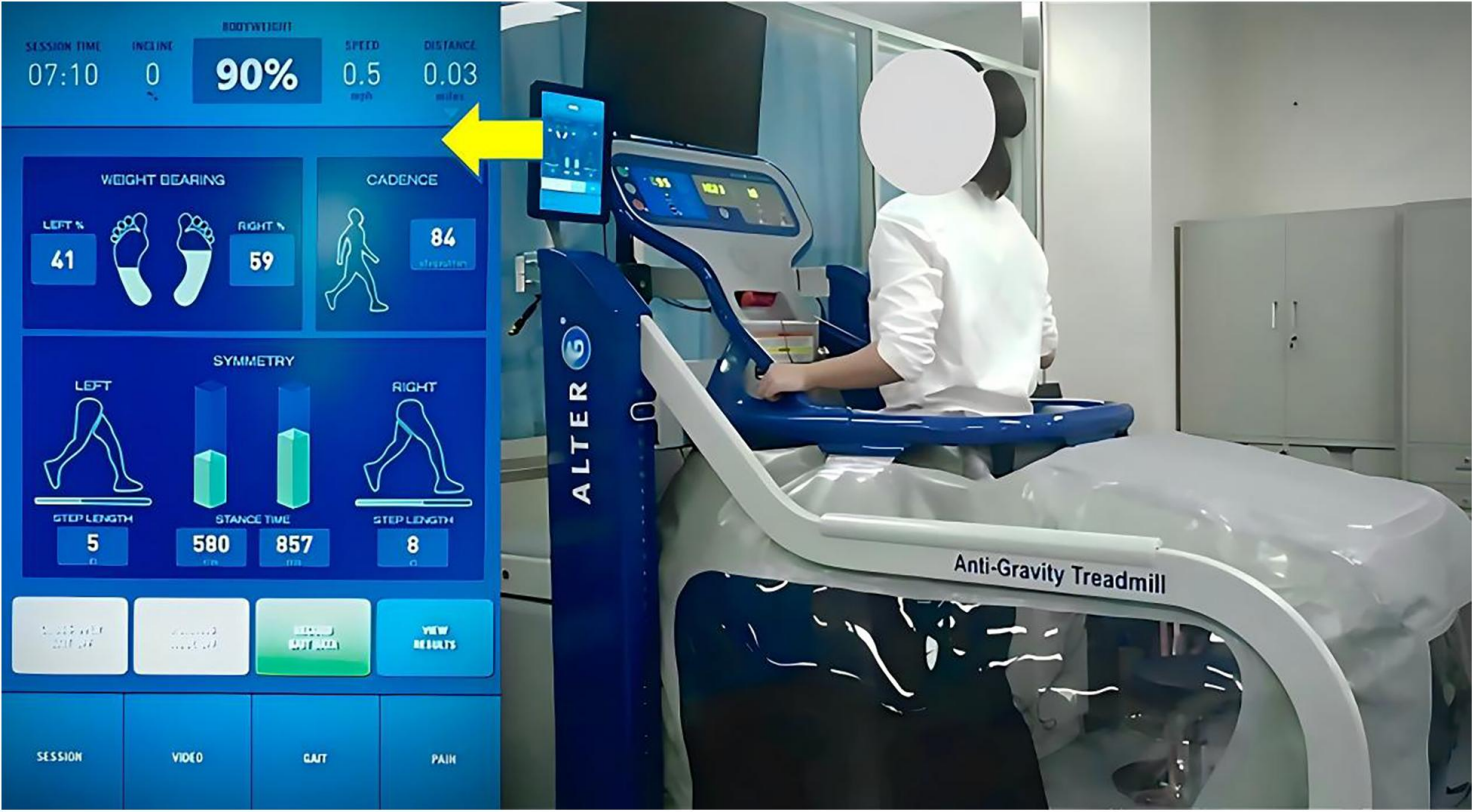
Gait training combined with weight loss and visual feedback.

The intervention will begin at 3 weeks post-surgery, with sessions conducted 3 times per week for 6 weeks. Each session will last 15–30 min (3 sets × 5–10 min/day, with 90-s rest intervals between sets). Each session will include a 5-min warm-up, followed by 5–10 min of stretching and ice application post-training. During the gait retraining, lower limb orthoses will be removed. Lower limb weight-bearing will be quantitatively and individually adjusted based on the postoperative week and the participant's condition (Table 1). During the gait retraining, the LBPP system's interactive screen will provide real-time feedback on bilateral lower limb weight-bearing and gait posture. The screen will display metrics such as ground reaction force on both feet, stance time, walking speed, step length, and steps per minute. Participants will be instructed to self-adjust their weight-bearing and gait patterns in real-time, using the visual feedback to achieve symmetry between limbs in ground reaction forces, step length, and stance time, with the goal of balanced bilateral weight-bearing throughout the gait cycle (Figure 3).

**Table 1:** Gait intervention protocol after ACLR.

Content	The first week	The second week	The third week	The fourth week	The fifth week	The sixth week
Loading degree	30%body weight	50%body weight	65%body weight	80%body weight	100%body weight	100%body weight
Time/time	15 min (3 × 5 min)	15 min (3 × 5 min)	15 min (3 × 5 min)	15 min (3 × 5 min)	1 (3 × 5 min)	15 min (3 × 5 min)
Rest/min	90 s	90 s	90 s	90 s	90 s	90 s
Trequency	3 times/week	3 times/week	3 times/week	3 times/week	3 times/week	3 times/week
Speed	Optional comfort speed (does not cause or aggravate pain)					

Stop Criteria:** The intervention will be terminated if the visual analog scale (VAS) pain score exceeds 4 points during a session, or if knee joint swelling increases by more than 10% compared to pre-session levels. VAS pain scores will be assessed before, during (at rest intervals), and immediately after gait retraining. Knee joint swelling will be assessed before and immediately after gait retraining.

#### Control group

Participants in the control group will perform the prescribed rehabilitation plan at home. Their rehabilitation status will be monitored through regular online follow-ups, and telephone guidance will be provided if issues arise. After 8 weeks, they will continue the home-based rehabilitation as per the protocol. At each postoperative follow-up visit, the next phase of home-based training will be explained, and precautions will be reviewed.

Both groups will follow the same conventional rehabilitation protocol, with sessions lasting 30 min each, 4–5 times per week. The detailed protocol is presented in Table 2.

**Table 2:** Postoperative rehabilitation protocol for ACLR patients.

Postoperative Time	Rehabilitation Content	Description	Number of Repetitions	Time (s)
Weeks 1–2	Ankle pump exercise	Maximal dorsiflexion for 5 s followed by plantarflexion for 5 s	120	5
	Quadriceps contraction	Forceful contraction of anterior thigh muscles for 5 s, then relax for 2 s	15	5
	Hamstring contraction	With knee fully extended, press down forcefully on a pillow placed under the heel	15	5
	Straight leg raise	Lift leg 15–60° off the bed, hold until exhaustion	10	10
	Knee extension exercise	Place a pillow under the heel, leaving the knee unsupported; relax muscles completely; mild posterior knee discomfort is normal	2	1,800
	Range of motion exercise	Seated beside bed with leg hanging; flex knee to target angle and hold for 10 min	1	300
	Weight shift training	Forward-backward and side-to-side directions	10	3
	Single-leg stance balance exercise	Stand straight, arms at sides, lift the affected foot and maintain balance	120	5
Week 3	Ankle pump exercise	Maximal dorsiflexion for 5 s followed by plantarflexion for 5 s	15	5
	Quadriceps contraction	Forceful contraction of anterior thigh muscles for 5 s, then relax for 2 s	15	5
	Hamstring contraction	With knee fully extended, press down forcefully on a pillow placed under the heel	10	10
	Straight leg raise	Lift leg 30–60° off the bed, hold until exhaustion	2	1,800
	Knee extension exercise	Place a pillow under the heel, leaving the knee unsupported; relax muscles completely; mild posterior knee discomfort is normal	2	1,800
	Range of motion exercise	Seated beside bed with leg hanging/seated pushing against wall; flex knee to target angle and hold	1	300
	Single-leg stance balance exercise	Stand straight, arms at sides, lift the affected foot and maintain balance	120	5
	Stepping exercise	Stand straight, hands at sides, march in place	1	180
	Walking exercise	Walk in front of a mirror, correcting abnormal gait patterns	1	180
Week 4	Straight leg raise	Lift leg 30–60° off the bed, hold until exhaustion	2	1,800
	Knee extension exercise	Place a pillow under the heel, leaving the knee unsupported; relax muscles completely; mild posterior knee discomfort is normal	2	1,800
	Range of motion exercise	Seated beside bed with leg hanging/seated pushing against wall; flex knee to target angle and hold	1	300
	Single-leg stance balance exercise	Stand straight, arms at sides, lift the affected foot and maintain balance	120	5
	High-knee marching exercise	Stand straight, hands at sides, march with high knees	1	180
	Walking exercise	Walk in front of a mirror, correcting abnormal gait patterns	1	300
Week 5	Resisted straight leg raise	With knee extended, lift leg 30–60° off the bed using an elastic band, hold until exhaustion	2	1,800
	Weighted knee extension exercise	Place a pillow under the heel, leaving the knee unsupported; relax muscles completely; a sandbag may be placed above the knee; mild posterior knee discomfort is normal	2	1,800
	Range of motion exercise	Seated, hold the knee to chest; flex to target angle and hold	1	300
	Single-leg stance balance exercise	Stand straight, arms at sides, lift the affected foot and maintain balance	120	5
	Step-up with healthy leg support	Healthy leg supports; affected leg rests on step, slowly perform stepping-up motion	15	3
	Reverse lunge with healthy leg	Stand straight with hands on hips, affected leg flexed at 90°, healthy leg steps back and kneels down	15	5
	High-knee marching exercise	Stand straight, hands at sides, march with high knees	1	300
	Walking exercise	Walk in front of a mirror, correcting abnormal gait patterns	2	300
Week 6	Resisted straight leg raise	With knee extended, lift leg 30–60° off the bed using an elastic band, hold until exhaustion	2	1,800
	Knee extension exercise	Place a pillow under the heel, leaving the knee unsupported; relax muscles completely; a sandbag may be placed above the knee; mild posterior knee discomfort is normal	2	1,800
	Range of motion exercise	Seated, hold the knee to chest; flex to target angle and hold	1	300
	Single-leg stance balance exercise	Stand straight, arms at sides, lift the affected foot and maintain balance	120	5
	Stepping exercise	Stand straight, hands at sides, march in place	1	180
	Step-down with affected leg support	Affected leg stands on step maintaining balance; healthy leg lowers down, touching the floor gently with the heel	15	3
	Reverse lunge with healthy leg	Stand straight with hands on hips, affected leg flexed at 90°, healthy leg steps back and kneels down	15	5
	Fast walking exercise	Walk in front of a mirror, correcting abnormal gait patterns	1	300
Week 7	Bilateral squat angle training	Symmetrical weight-bearing on both lower limbs; train knee flexion angle and lower limb strength through squatting	1	900
	Backward step-up with affected leg support	Affected leg performs single-leg support; healthy leg steps backward onto a step	20	3
	Supine weighted straight leg raise	Supine, lift straight leg upward with resistance provided by a sandbag	15	10
	Prone weighted hamstring curl	Prone, flex the knee against resistance provided by a sandbag	15	10
	Wall squat exercise	Squat against a wall in a pain-free range; ensure knees do not extend beyond toes	5	40
	Forward lunge with affected leg	Stand straight with hands on hips, healthy leg flexed at 90°, affected leg steps back and kneels down	5	5
	Fast walking exercise	Walk in front of a mirror, correcting abnormal gait patterns	2	300
Week 8	Bilateral squat angle training	Symmetrical weight-bearing on both lower limbs; train knee flexion angle and lower limb strength through squatting	1	900
	Supine weighted straight leg raise	Supine, lift straight leg upward with resistance provided by a sandbag	15	10
	Prone weighted hamstring curl	Prone, flex the knee against resistance provided by a sandbag	15	10
	Wall squat exercise	Squat against a wall in a pain-free range; ensure knees do not extend beyond toes	5	40
	Fast walking exercise	Walk in front of a mirror, correcting abnormal gait patterns	2	300
Weeks 9–3 months	Wall squat exercise	Squat against a wall in a pain-free range; ensure knees do not extend beyond toes	5	40
	Bilateral countermovement jump	Squat bilaterally then jump upward and land	15	3
	Single-leg countermovement jump (affected side)	Squat on affected leg then jump upward and land	10	3
	Standing resisted leg raise with elastic band	Standing, perform leg raises in multiple directions against elastic band resistance	15	10
	Fast walking exercise	Walk in front of a mirror, correcting abnormal gait patterns	2	300

### Statistical analysis

The primary analysis will employ a mixed-effects model to account for within-subject correlations across repeated measures during the 2-year follow-up and to handle potentially missing data at random. All statistical tests will be performed with a significance level set at *α* = 0.05. A repeated-measures two-way analysis of variance (ANOVA) will be used to evaluate the interaction effects between group (experimental vs. control) and time (baseline, 3 months, 6 months, 1 year, and 2 years) on cartilage biochemical characteristics derived from functional MRI (T1*ρ*/T2 values) and on gait parameters. For multiple comparisons, the Bonferroni correction will be applied. For EMG data, repeated-measures ANOVA will be used to assess the differences in muscle activation patterns before and after gait retraining, thereby exploring the neuromuscular control mechanisms underlying the effects of gait retraining on gait biomechanics. To control the false discovery rate (FDR) when comparing MRI parameters across 10 cartilage subregions and multiple time points, the FDR method will be applied with the threshold set at 0.05. Missing data will be assumed to be missing at random (MAR) and will be addressed using the mixed-effects model without imputation or deletion. For the analysis populations, the primary analysis will follow the intention-to-treat (ITT) principle, including all participants as originally randomized. Sensitivity analysis will be performed using a per-protocol (PP) set, including only participants who completed the full intervention and all follow-up assessments. Effect sizes will be reported as partial eta-squared (*η*^2^). Outliers, defined as values exceeding ±3 standard deviations from the group mean, will be excluded and reanalyzed in a sensitivity analysis.

### Statement of ethics and consent to participate

The National Bioethics Committee's Review Board granted ethical approval for this research with human participants. Written informed consent will be obtained from all study participants.

## Discussion

Current standardized rehabilitation protocols following ACLR primarily focus on restoring muscle strength, range of motion, and neuromuscular coordination. However, even if these indicators return to normal, abnormal gait biomechanical characteristics still cannot be completely corrected, and specific GRT should be added to restore gait. Therefore, this study aims to address the gap in incorporating specific GRT into post-ACLR rehabilitation programs. By investigating whether GRT can optimize standard rehabilitation, we hope to provide a strategy for preventing or delaying the occurrence and progression of cartilage degeneration. This has important clinical implications for helping athletes and active individuals restore normal gait and prevent secondary injuries.
